# Associations between visceral adipose index and stress urinary incontinence among US adult women: a cross-sectional study

**DOI:** 10.1007/s00345-023-04667-7

**Published:** 2023-11-03

**Authors:** Haigang Pang, Yuxin Yin, Juan Xue, Xi Chen, Jian Pang, Jinping Zhang, Yi Sun

**Affiliations:** 1 Department of Urology, No.971 Hospital of the PLA Navy, Qingdao, 266071 China; 2 Department of Nursing, No.971 Hospital of the PLA Navy, Qingdao, 266071 China

**Keywords:** Obesity, Stress urinary incontinence, Visceral fat, Visceral adiposity index

## Abstract

**Objective:**

Visceral adipose index (VAI) is a novel parameter for the evaluation of visceral obesity. The present study aimed to investigate the association between VAI levels and stress urinary incontinence (SUI) in a nationally representative population.

**Materials and methods:**

The National Health and Nutrition Examination Survey (NHANES) women population aged > 20 years were analyzed from 2001 to 2018. SUI was determined by self-reported questions. VAI was calculated using physical examination data and laboratory tests. Survey-weighted logistic regression models were used to analyze the correlation between SUI and VAI.

**Results:**

The final analysis included 9709 women. Among them, 4032 (41.53%) were any SUI, 1130 (11.64%) were at least weekly SUI, and 506 (5.21%) were at least daily SUI. In multivariate analysis, the odds ratio (OR) for overall SUI increased slightly after full adjustment (OR 1.06, 95% CI 1.03–1.10, P = 0.001). Similar results were observed in weekly (OR 1.04, 95% CI 1.00–1.08, P = 0.0327) and daily (OR 1.04, 95% CI 1.00–1.09, P = 0.0702) SUI. The analysis of VAI categorized showed an increased OR of any, weekly, and daily SUI in the highest compared to the lowest tertile (OR 1.44, 95% CI 1.26–1.65, P < 0.0001 for trend, OR 1.38, 95% CI 1.07–1.78, P = 0.0153 for trend, OR 1.33, 95% CI 0.94–1.87, P = 0.094 for trend).

**Conclusion:**

This study revealed a significant association between SUI and VAI among US adult women. VAI is an easily applicable index for the evaluation of visceral fat dysfunction, which might be useful for the calculation of SUI risk.

**Supplementary Information:**

The online version contains supplementary material available at 10.1007/s00345-023-04667-7.

## Introduction

Stress urinary incontinence (SUI) is defined by the International Continence Society (ICS) as the complaint of any involuntary loss of urine on effort or physical exertion (e.g., sporting activities) or on sneezing or coughing [1]. The overall prevalence of SUI (when defined as any symptoms in the previous year) is about 40–46% among adult women in the USA [2, 3]. SUI significantly impairs the quality of life and contributes to a significant financial burden. Furthermore, with the aging community and the pursuit of a better quality of life, these costs are expected to increase in the next few decades [4].

Being overweight and obese have been identified as independent risk factors for the development of urinary incontinence [5], even in young to mid-aged women [6]. The increasing rates of obesity could be attributed to excessive triglyceride (TG) storage in white adipose tissue (WAT) [7], the core factor of the obesity pandemic. WAT’s response to excess calories has an effect on every organ system, with profound effects on morbidity and mortality worldwide. WAT is commonly separated into visceral fat and subcutaneous fat, which confers negative and neutral or positive metabolic effects respectively. The current assessments across adult and pediatric populations often use body mass index (BMI) exclusively, which requires adjustments for age, sex, and genetic and ethnic backgrounds [8]. BMI cannot provide information about WAT distribution or the predispositions of specific depots for distinctive pathophysiology or the likelihood of a response to targeted therapies. Thus, large studies of epidemiologic trends beyond BMI and incorporating other estimates of the distribution of WAT depots, including waist circumference [9], are essential to understand the role of WAT distribution in obesity-related diseases. A previous study showed that the identification of a routinely applicable indicator for the evaluation of visceral adipose function, with higher sensitivity and specificity than classical parameters (such as waist circumference, BMI, and lipids), could be useful for cardiometabolic risk assessment [10]. Subsequently, a model of adipose distribution (MOAD) was constructed. To correct MOAD for fat function, TG and high-density lipoprotein (HDL) levels were introduced in the formula. The visceral adipose index (VAI) was used in this formula for cardiometabolic risk assessment. A previous small sample study [11] showed that the VAI levels were statistically higher in women with SUI.

However, there is limited evidence regarding the potential association between VAI and SUI. Therefore, the present study aimed to investigate the cross-sectional association between VAI and SUI using the National Health and Nutrition Examination Survey (NHANES).

## Material and methods

### Study population

**Fig. 1:**
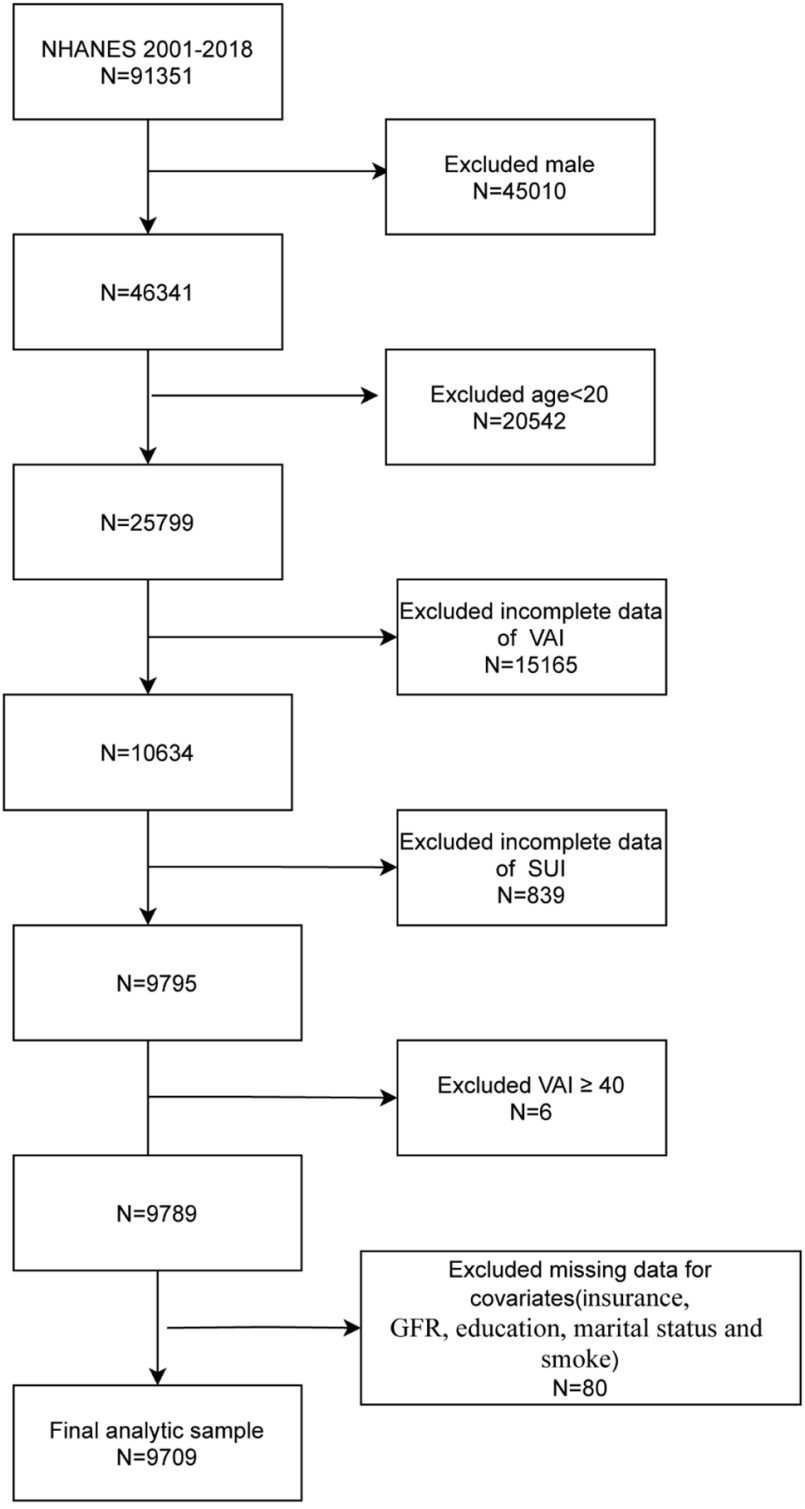
Study flow chart. VAI-visceral adipose index; SUI-Stress Urinary Incontinence

NHANES is a nationally representative cross-sectional survey administered by the National Center for Health Statistics to assess the health and nutritional status of adults and children in the United States. To increase the sample size, we used the NHANES datasets from 2001 to 2018. The study population was limited to women ≥ 20-years-old. Next, we excluded the study participants with missing information: 15,165 for VAI, 839 for SUI, 80 for covariates (insurance, GFR, education, marital status, and smoking), and 6 with VAI > 40 (see Fig. 1).

### SUI

The primary outcome, SUI, was determined by self-reporting: “During the past 12 months, have you leaked or lost control of even a small amount of urine with activity like coughing, lifting, or exercise?” SUI severity was characterized by the response to the question, “How frequently does this occur?” At least weekly SUI and at least daily SUI were characterized as variables independent of overall SUI.

### VAI

NHANES researchers collected anthropometric data (i.e., BMI and waist circumference), biochemical data [i.e., high-density lipoprotein (HDL)-cholesterol, and fasting TG] that were used to calculate the VAI for females using this formula:

$${\text{VAI}}:{\text{WC}}/\left[ {36.58 + \left( {1.89 \times {\text{BMI}}} \right)} \right] \times {\text{TG}}/0.81 \times 1.52/{\text{HDL}}{.}$$

WC: waist circumference, BMI: body mass index, TG: triglyceride, HDL: high-density lipoprotein.

### Covariates

Demographic information, including age, self-reported race/ethnicity, and marital status, was used for adjustment. The education level was categorized as less or greater than a high school. The response to the question determined health insurance coverage. “Are you covered by health insurance or some other kind of health care plan?” The poverty income ratio (PIR), which uses the ratio of income to the family’s poverty threshold set by the US Census Bureau, serves as an indicator of socioeconomic status. Alcohol and caffeine intake was obtained from 24-h dietary recall interviews. Physical activity was assessed by interview using a questionnaire. Each of the activities was awarded an energy expenditure on the metabolic equivalent (MET) scores. The MET-min per week of each activity was calculated by multiplying the standard MET value of each activity by the total number of minutes per week of each activity; then, the total MET-min per week was calculated as the sum of MET-min per week of each activity. Excluding diabetes and hypertension, assessed separately, other chronic diseases were combined to establish a comorbidity index. Parity was defined as the number of total cesarean and vaginal deliveries. Smoking status was categorized as never, former, and current. The glomerular filtration rate (GFR) was determined using the Modification of Diet in Renal Disease (MDRD) Study equation based on the laboratory data on creatinine [12]. GFR was dichotomized as < 60 and ≥ 60 mL/min/1.73 m^2^ to define chronic kidney disease. Depression was assessed during a private interview in the mobile examination centers using the validated Patient Health Questionnaire-9 (PHQ-9). The PHQ-9 yields scores from 0 to 27 and scores ≥ 10 are used to define major depression.

### Statistical analysis

All statistical analyses were performed using R packages (http://www.r-project.org) and EmpowerStats (www.empowerstats.com, X&Y Solutions Inc., Boston, MA, USA). All analyses considered sample weights and strata and cluster design of the complex NHANES design [13]. The categorical variables were expressed as survey-weighted percentage [95% confidence interval (CI)] by survey-weighted chi-square test (syytable), and continuous variables were presented as survey-weighted mean (95% CI) by survey-weighted linear regression (svyglm).

Three independent logistic models were constructed to evaluate the associations between VAI and the odds of overall SUI, at least weekly SUI, or at least daily SUI, as reported by participants. All covariates were chosen based on known or suspected confounders of the correlation between VAI and SUI in women. The final model adjusted for age, race, marital status, education, insurance, poverty income ratio (PIR), caffeine intake, physical activity, diabetes, hypertension, Glomerular filtration rate (GFR), smoke, parity, depression and comorbidity index. The significance of trends across these three groups was calculated and reported with the p-value. The results of these analyses are presented as the OR and 95% CI, and P < 0.05 was considered significant.

### Subgroup

Associations between SUI and VAI were analyzed in different subgroups based on the covariates with separate logistic regression models for each subgroup. The interactions in subgroups, tested by including a multiplicative term in the logistic model, were denoted by P values.

### Sensitivity

Dummy variables were used to indicate the missing covariate values. We also used multiple imputations as sensitivity analysis, based on five replicates and a chained equation approach method in the R MI procedure to account for missing data.

## Results

### Baseline characteristics

A total of 9,709 women > 20-years-old were included in this study. Among them, 4032 (41.53%) reported any SUI, 1130 (11.64%) reported at least weekly SUI, and 506 (5.21%) reported at least daily SUI. The baseline characteristics of the study participants by VAI tertiles are shown in Table 1. The median baseline VAI was 1.55, with an interquartile range of 0.94–2.57. Those in the highest tertiles were more likely to be older, non-Hispanic White, Mexican American, separated or divorced or widowed, current smokers, less than high school educated, uninsured, have lower PIR, alcohol, caffeine intake, and eGFR, less physical activity, higher proportion of hypertension and diabetes, and a greater number of parity and comorbidities.

**Table 1:** Basic characteristics according to VAI tertiles

	Lowest tertile 0.10—1.11	Middle tertile 1.11—2.13	Highest tertile 2.13–37.66	P value
Age, mean (95%CI), years	44.52 (43.73,45.32)	47.78 (46.92,48.65)	51.27 (50.58,51.96)	< 0.0001
Race/Ethnicity, percentage (95% CI)				< 0.0001
Mexican American	5.30 (4.36,6.43)	8.21 (7.00,9.60)	9.46 (7.98,11.18)	
Other Hispanic	4.79 (3.77,6.08)	5.06 (4.18,6.12)	5.69 (4.38,7.37)	
Non-Hispanic White	66.44 (63.53,69.23)	69.20 (66.44,71.82)	71.26 (68.33,74.01)	
Non-Hispanic Black	16.12 (14.21,18.22)	10.98 (9.56,12.58)	6.73 (5.78,7.82)	
Other Race	7.35 (6.22,8.67)	6.56 (5.57,7.70)	6.86 (5.64,8.33)	
Marital status, percentage (95% CI)				< 0.0001
Never married	19.63 (17.78,21.62)	15.07 (13.32,17.01)	11.00 (9.58,12.59)	
Married or living with partner	61.23 (58.70,63.70)	61.70 (59.45,63.91)	62.28 (59.98,64.52)	
Separated/divorced/Widowed	19.24 (17.47,21.13)	23.16 (21.29,25.14)	26.75 (24.92,28.65)	
Education less than high school, percentage (95% CI)	45.82 (43.18,48.48)	53.07 (50.44,55.68)	59.92 (57.29,62.49)	< 0.0001
Insurance, percentage (95% CI)	86.76 (85.18,88.19)	84.04 (82.26,85.68)	83.36 (81.60,84.99)	0.0212
PIR, mean (95%CI), mg/d	2.99 (2.90,3.07)	2.73 (2.62,2.84)	2.51 (2.41,2.61)	< 0.0001
Missing PIR, percentage (95% CI)	5.77 (4.90,6.78)	5.89 (4.94,7.02)	6.37 (5.41,7.48)	0.6462
Alcohol intake, mean (95%CI), g/d	0.98 (0.87,1.09)	0.66 (0.57,0.76)	0.48 (0.37,0.58)	< 0.0001
Missing Alcohol intake, percentage (95% CI)	9.38 (8.10,10.85)	8.05 (6.94,9.31)	8.06 (6.72,9.64)	0.2475
Caffeine intake, mean (95%CI), mg/d	133.92 (125.65,142.20)	140.82 (132.14,149.51)	150.60 (142.69,158.50)	0.0074
Missing Caffeine intake, percentage (95% CI)	9.38 (8.10,10.85)	8.05 (6.94,9.31)	8.06 (6.72,9.64)	0.2475
Physical activity, mean (95%CI), MET-min/week	2395.03 (2182.22,2607.84)	1909.33 (1744.08,2074.57)	1605.04 (1442.21,1767.87)	< 0.0001
Missing physical activity, percentage (95% CI)	18.88 (17.19,20.69)	24.62 (22.89,26.43)	31.76 (29.64,33.96)	< 0.0001
Diabetes, percentage (95% CI)				< 0.0001
Yes	5.61 (4.82,6.51)	12.64 (11.26,14.17)	26.55 (24.65,28.53)	
No	92.97 (92.00,93.84)	84.76 (83.10,86.29)	71.37 (69.35,73.32)	
Not recorded	1.50 (1.13,1.99)	2.98 (2.41,3.67)	2.83 (2.24,3.57)	
Hypertension, percentage (95% CI)	23.02 (21.08,25.08)	32.51 (30.63,34.45)	45.40 (43.18,47.63)	< 0.0001
GFR, percentage (95% CI), mL/min/1.73 m^2^				< 0.0001
< 60	6.04 (5.09,7.16)	8.07 (7.01,9.28)	12.96 (11.35,14.75)	
≥ 60	93.96 (92.84,94.91)	91.93 (90.72,92.99)	87.04 (85.25,88.65)	
Smoke				< 0.0001
Never	67.00 (64.74,69.18)	60.38 (58.03,62.69)	52.41 (49.98,54.83)	
Former	20.06 (18.19,22.07)	21.03 (18.85,23.39)	22.99 (20.90,25.23)	
Now	12.94 (11.60,14.41)	18.59 (16.68,20.67)	24.60 (22.67,26.63)	
Parity, mean (95% CI)	1.69 (1.61,1.76)	2.00 (1.92,2.09)	2.21 (2.13,2.29)	< 0.0001
Missing parity, percentage (95% CI)	27.59 (25.41,29.89)	19.72 (17.70,21.91)	14.92 (13.09,16.95)	< 0.0001
PHQ-9, percentage (95% CI)				< 0.0001
< 10	77.20 (75.35,78.94)	71.81 (69.37,74.13)	66.01 (63.04,68.86)	
≥ 10	5.52 (4.69,6.48)	6.01 (4.96,7.27)	10.23 (8.83,11.82)	
Not recorded	17.29 (15.67,19.04)	22.18 (20.07,24.45)	23.76 (21.08,26.66)	
Comorbidity index, percentage (95% CI)				< 0.0001
0	65.71 (63.43,67.92)	56.12 (53.93,58.29)	40.43 (38.09,42.81)	
1	16.17 (14.50,18.00)	20.39 (18.76,22.13)	24.41 (22.30,26.65)	
≥ 2	18.12 (16.44,19.92)	23.49 (21.56,25.53)	35.16 (32.94,37.46)	
Stress incontinence overall, percentage (95% CI)	35.41 (33.26,37.63)	42.45 (40.19,44.74)	52.17 (49.77,54.56)	< 0.0001
Stress Incontinence Weekly, percentage (95% CI)	19.83 (17.17,22.79)	26.44 (23.36,29.76)	29.81 (27.03,32.76)	< 0.0001
Stress incontinence daily, percentage (95% CI)	8.55 (6.83,10.64)	11.15 (9.03,13.69)	13.65 (11.51,16.11)	0.0072

Data in the table: For continuous variables: survey-weighted mean (95% CI), P value was by survey-weighted linear regression (svyglm)

For categorical variables: survey-weighted percentage (95% CI), P-value was by survey-weighted Chi-square test (svytable)

*VAI* visceral adipose index, *PIR* poverty income ratio, *GFR* Glomerular filtration rate; *PHQ-9* Patient Health Questionnaire-9

### VAI and SUI associations

The ORs for overall, at least weekly, and at least daily SUI by tertile categories and one-unit increment in VAI are shown in Table 2. Univariate analyses (model 1) showed that VAI is associated with a significant increase in the incidence of overall, at least weekly, and at least daily SUI, after adjusting for age, race, marital status, education, PIR, and insurance. This was also true in Model 2.

**Table 2:** Multivariable adjusted models of VAI associations with overall, at least weekly and at least daily SUI

	Tertile of VAI			P value for trend	VAI continuous
	Low 0.10–1.11	Middle 1.11–2.13	High 2.13–37.66		
Overall SUI (OR, 95% CI, P)					
Model 1	Ref	1.30 (1.15, 1.46) < 0.0001	1.98 (1.74, 2.25) < 0.0001	< 0.0001	1.13 (1.08, 1.18) < 0.0001
Model 2	Ref	1.18 (1.04, 1.34) 0.0094	1.64 (1.44, 1.87) < 0.0001	< 0.0001	1.09 (1.05, 1.13) < 0.0001
Model 3	Ref	1.13 (0.99, 1.28) 0.0729	1.44 (1.26, 1.65) < 0.0001	< 0.0001	1.06 (1.03, 1.10) 0.0010
Weekly SUI (OR, 95% CI, P)					
Model 1	Ref	1.60 (1.28, 2.02) 0.0001	2.41 (1.92, 3.02) < 0.0001	< 0.0001	1.11 (1.06, 1.15) < 0.0001
Model 2	Ref	1.39 (1.10, 1.75) 0.0065	1.82 (1.45, 2.27) < 0.0001	< 0.0001	1.08 (1.04, 1.12) < 0.0001
Model 3	Ref	1.23 (0.96, 1.57) 0.1023	1.38 (1.07, 1.78) 0.0158	0.0153	1.04 (1.00, 1.08) 0.0327
Daily SUI (OR, 95%CI, P)					
Model 1	Ref	1.49 (1.04, 2.14) 0.0298	2.47 (1.81, 3.35) < 0.0001	< 0.0001	1.10 (1.06, 1.14) < 0.0001
Model 2	Ref	1.23 (0.85, 1.77) 0.2716	1.73 (1.26, 2.38) 0.0010	0.0007	1.08 (1.04, 1.12) 0.0002
Model 3	Ref	1.09 (0.75, 1.58) 0.6594	1.33 (0.94, 1.87) 0.1082	0.0940	1.04 (1.00, 1.09) 0.0702

Model1: adjust for: None. Model2: adjust for: age; race; marital status; education; PIR; insurance. Model3: adjust for: age; race; marital status; education; insurance; PIR; caffeine intake; physical activity; diabetes; hypertension; GFR; smoke; parity; depression; comorbidity index

*VAI* visceral adipose index, *SUI* stress urinary incontinence, *PIR* poverty income ratio, *GFR* glomerular filtration rate, *PHQ-9* Patient Health Questionnaire-9, *CI* confidence interval, *Ref* reference, *OR* odds ratio

After adjustment for all covariates, the OR for overall SUI was slightly increased (OR 1.06, 95% CI 1.03–1.10, P = 0.001). Similar results were observed in weekly (OR 1.04, 95% CI 1.00–1.08, P = 0.0327) and daily (OR 1.04, 95% CI 1.00–1.09, P = 0.0702) SUI. Analysis with VAI categorized as tertiles revealed significantly increased odds of any and weekly SUI in the highest compared to the lowest tertiles (OR 1.44, 95% CI 1.26–1.65, P < 0.0001 for trend, OR 1.38, 95% CI 1.07–1.78, P = 0.0153 for trend) in the full adjustment model. However, in daily SUI, the association with VAI was not statistically significant while comparing the participants in the highest *vs*. the lowest VAI group tertiles (OR 1.33, 95% CI 0.94–1.87, P = 0.094 for trend).

### Subgroup analysis

In the stratified analyses for SUI with a one-unit increment in VAI, stronger positive associations were found in those with eGFR ≥ 60 mL/min/1.73 m^2^ (P for interaction = 0.0043) (Supplementary Table S1). No significant interactions were detected for other covariates on the associations between SUI and VAI.

### Sensitivity

A post-hoc sensitivity analysis using multiple imputations for accounting for missing data on PIR (8.03%), alcohol intake (10.13%), caffeine intake (10.13%), physical activity (30.16%), diabetes (5.02%), parity (18.43%), and PHQ-9 (21.45%) showed similar results compared to the fully adjusted models in Table 2. The pooled ORs of five imputations for overall, at least weekly, and at least daily SUI were 1.07 (95% CI 1.03–1.11), 1.05 (95% CI 1.01–1.09), 1.06 (95% CI 1.02–1.10), respectively (Table 3).

**Table 3:** ORs of five multiple imputation data and pooled OR (OR, 95%CI)

			MI 1	MI 2	*MI 3*	MI 4	MI 5	Pooled OR
Overall SUI		VAI continuous	1.07 (1.03, 1.11)	1.07 (1.03, 1.11)	1.07 (1.03, 1.11)	1.07 (1.03, 1.11)	1.07 (1.03, 1.10)	1.07 (1.03, 1.11)
	Tertile of VAI	Low	Ref	Ref	Ref	Ref	Ref	Ref
		Middle	1.15 (1.01, 1.31)	1.16 (1.02, 1.32)	1.16 (1.02, 1.32)	1.16 (1.02, 1.31)	1.15 (1.01, 1.31)	1.16(1.02,1.32)
		High	1.47 (1.28, 1.69)	1.48 (1.29, 1.70)	1.48 (1.29, 1.70)	1.46 (1.27, 1.67)	1.47 (1.28, 1.68)	1.47(1.28,1.69)
Weekly SUI		VAI Continuous	1.06 (1.02, 1.10)	1.05 (1.01, 1.09)	1.05 (1.02, 1.09)	1.05 (1.02, 1.09)	1.05 (1.02, 1.09)	1.05(1.01,1.09)
	Tertile of VAI	Low	Ref	Ref	Ref	Ref	Ref	Ref
		Middle	1.31 (1.02, 1.67)	1.31 (1.03, 1.66)	1.32 (1.04, 1.68)	1.31 (1.03, 1.67)	1.31 (1.03, 1.67)	1.31(1.03,1.67)
		High	1.48 (1.05, 2.08)	1.51 (1.18, 1.94)	1.54 (1.20, 1.99)	1.51 (1.17, 1.94)	1.52(1.18,1.96)	1.51(1.15,1.99)
Daily SUI		VAI Continuous	1.06 (1.02, 1.10)	1.06 (1.02, 1.10)	1.06 (1.02, 1.10)	1.06 (1.01, 1.10)	1.06 (1.02, 1.10)	1.06(1.02,1.10)
	Tertile of VAI	Low	Ref	Ref	Ref	Ref	Ref	Ref
		Middle	1.17 (0.81, 1.71)	1.17 (0.81, 1.68)	1.18 (0.81, 1.71)	1.16 (0.80, 1.68)	1.18 (0.81, 1.72)	1.17(0.81,1.70)
		High	1.48 (1.05, 2.08)	1.46 (1.05, 2.03)	1.48 (1.06, 2.06)	1.43(1.48,2.00)	1.47(1.05, 2.07)	1.46(1.07,1.99)

*VAI* visceral adipose index, *SUI* stress urinary incontinence, *CI* confidence interval, *Ref* reference, *OR* odds ratio

## Discussion

In this cross-sectional study, a nationally representative sample of women in the United States, ≥ 20-years-old, overweight and obese, as indicated by higher VAI scores, was associated with an increased likelihood of SUI after adjusting for demographic and health-related covariates. Additionally, this risk was further increased among participants with eGFR ≥ 60 mL/min/1.73m^2^.

The association between SUI and obesity has been shown by some studies [14, 15]. Also, the impact of metabolic syndrome and dyslipidemia on urinary incontinence development has been examined [16, 17]. A systematic review by Hunskaar et al. showed that being overweight and obese is a strong risk factor for urinary incontinence [17]. The study suggested that intra-abdominal pressure increases with excess body weight, bladder pressure, and urethral mobility, leading to SUI and also exacerbating detrusor overactivity. Moreover, the prolonged effect on the pelvic musculature, nerve supply, and supporting structures due to chronic strain may cause pelvic floor muscle weakness and negatively impact pelvic organ function.

It has been hypothesized that oxidative stress related to adipose tissue increases the prevalence and severity of urinary incontinence by altering collagen metabolism. Visceral adipose tissue is an endocrine organ. In overweight and obese people, the secretion of inflammatory cytokines and factors, such as tumor necrosis factor-alpha (TNF-a) and interleukin-6 (IL-6), is unbalanced [18]. Leptin activates the nicotinamide adenine dinucleotide phosphate (NADPH) oxidase, stimulates the production of reactive oxygen species (such as hydrogen peroxide, H_2_O_2_), also increases oxidative stress in obesity [19]. Liu et al. showed that exogenous H_2_O_2_ has a bidirectional regulatory effect on collagen metabolism [20]. After incubation with human uterosacral ligament fibroblasts in vitro for 24 h, lower concentrations of H_2_O_2_ stimulated the anabolism of collagen type 1 alpha 1 (COL1A1), while higher concentrations of H_2_O_2_ promoted catabolism. Notably, with the increase in oxidative stress, the upregulation of transforming growth factor-beta 1 (TGF-b1) and proteolytic enzymes, such as matrix metalloproteinase-2 (MMP-2), promotes collagen catabolism. The results showed that oxidative stress leads to the disorder of collagen metabolism in human pelvic fibroblasts. Therefore, the physiological and biochemical stress of obesity on the neuromuscular system of the pelvic floor might lead to the development of urinary incontinence [21].

BMI is applied to define overweight and obesity in epidemiological studies. However, it is a poor estimate of overall obesity because it cannot distinguish between lean and fat mass and the types of adipose tissue depots, such as visceral and subcutaneous storage [22]. Conversely, several studies have shown that visceral adipose tissue and low lean mass, independent of BMI, are associated with a high risk of SUI [23]. In summary, additional studies are required to understand the biological basis of the obesity paradox [24]. Relying only on BMI to assess the prevalence of obesity could hinder future interventions aimed at the prevention and control of SUI. As an index of adipose tissue dysfunction, VAI has gradually been used as a surrogate marker associated with all metabolic syndrome factors [10]. It indirectly reflects non-classical risk factors, such as altered production of adipocytokines, increased lipolysis, and plasma free fatty acids, which are independent of BMI, WC, TG, and HDL, respectively. On the other hand, obesity leads to insulin resistance, which in turn adversely affects the lipid ratio, resulting in lower HDL cholesterol and higher triglycerides and LDL cholesterol in the blood. These suboptimal cholesterol ratios may lead to the accumulation of atheromatous deposits in the bladder wall, resulting in bladder wall ischemia, urothelial dysfunction, and increased risk of SUI [11]. Therefore, VAI may be a valuable indicator of fat distribution and function. Some recent studies confirmed that VAI is significantly associated with SUI [9, 23]. In the current study, a 44% increase was noted in the incidence of SUI in the highest compared to the lowest VAI group.

Unlike the previous studies, we used a large sample to evaluate the association between VAI and SUI and adjusted for critical variables based on previous studies and expert recommendations. We also performed sensitivity analyses, including different SUI severities as outcome variables. On the other hand, multiple imputations for covariates did not alter the association between SUI and VAI, suggesting that our results are robust and reliable.

Nevertheless, the present study had some limitations. The current data were obtained from a cross-sectional and observational study. Therefore, it was impossible to infer the causal correlation and calculate the incidence of SUI. Since most data were collected in the form of questionnaires, there may be a recall bias. Another limitation was determining the subjects treated with SUI because this information would help determine the limitations of urological treatment. Furthermore, the respondents could not accurately distinguish between UUI and SUI. However, previous studies reported that the accurate answer to the incontinence question was similar to that in the NHANES questionnaire. Due to the limitations of NHANES, we did not conduct a strict severity grading of SUI. The categorization of different SUI in this article is merely part of a sensitivity analysis, thus the conclusions cannot be linked to the severity of SUI. Moreover, this study was only conducted among women, and cannot be extrapolated to the male population. Finally, missing data led to the deviation in our sample; however, only a small subgroup had missing data. Next, we conducted data imputation as a sensitivity analysis to ensure the robustness of the results.

## Conclusions

Our study showed a significant association between VAI and SUI in a large, diverse, and nationally representative sample of women after meaningful adjustment. VAI is a simple and easy new index to evaluate visceral fat dysfunction and a useful index to assess and calculate the risk of SUI. Future prospective studies are required to further strengthen our findings and explore the potential pathophysiological mechanisms, thus allowing us to understand the occurrence and development of SUI and evaluate its potential therapeutic implications.

### Supplementary Information

Below is the link to the electronic supplementary material.

The results of stratified analyses are provided in the supplementary table S1 and supplementary Fig. S1 (JPG 599 KB)

Supplementary file2 (DOCX 19 KB)

## Data Availability

All data generated or analyzed during this study are included in this published article [and its supplementary information files].
