# Supplementary figures and images for: Associations between visceral adipose index and stress urinary incontinence among US adult women: a cross-sectional study
Source: World J Urol. 2023 Nov 3;41(12):3671–8. doi: 10.1007/s00345-023-04667-7 (PMC10693499; doi:10.1007/s00345-023-04667-7)

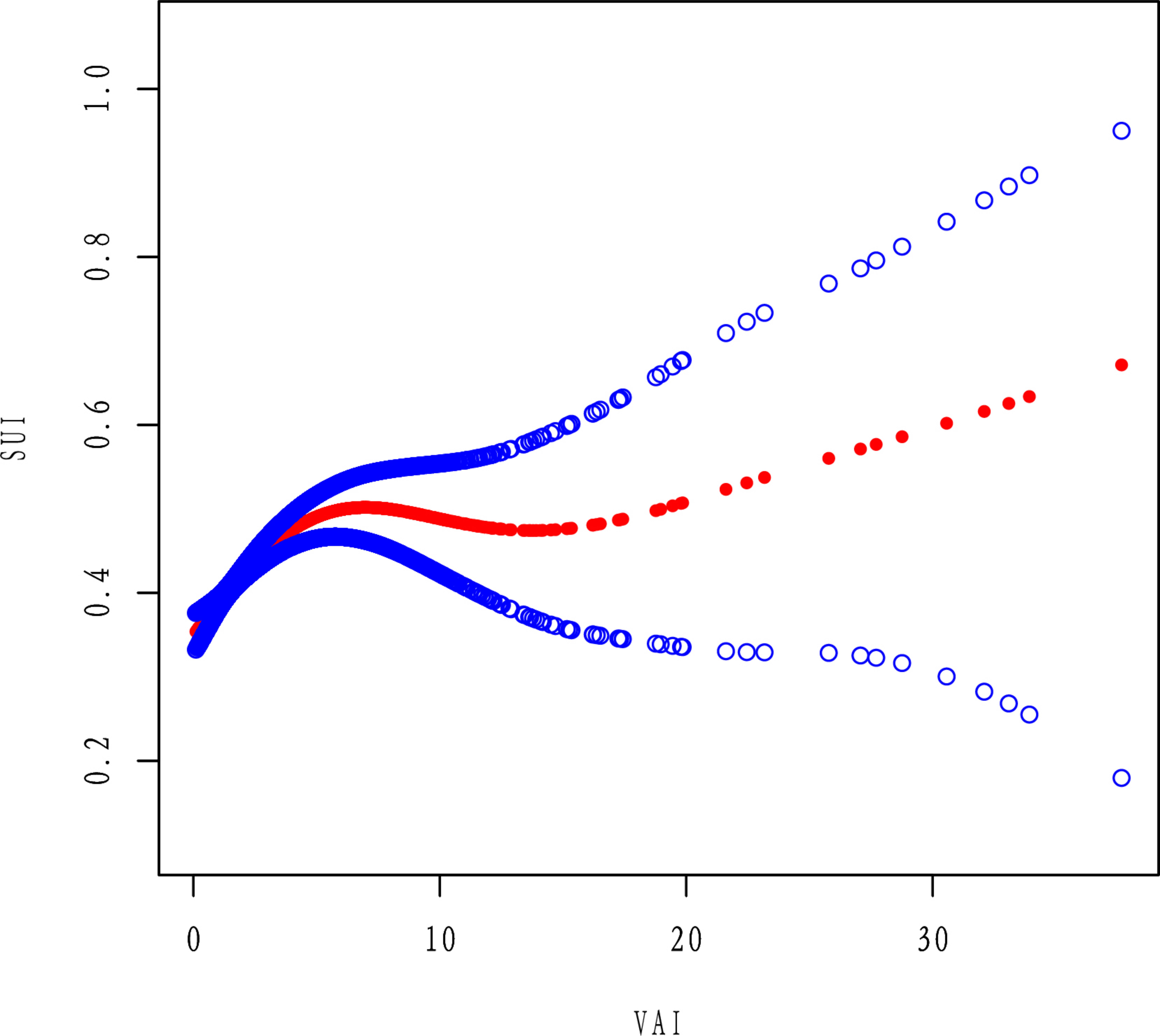

Supplement: Supplementary file 1 — The results of stratified analyses are provided in the supplementary table S1 and supplementary Fig. S1 (JPG 599 KB) [file 345_2023_4667_MOESM1_ESM.jpg]
