# Supplementary material for: Associations between visceral adipose index and stress urinary incontinence among US adult women: a cross-sectional study
Source: World J Urol. 2023 Nov 3;41(12):3671–8. doi: 10.1007/s00345-023-04667-7 (PMC10693499; doi:10.1007/s00345-023-04667-7)
Supplement: Supplementary file 2 — Supplementary file2 (DOCX 19 KB) [file 345_2023_4667_MOESM2_ESM.docx]

**Supplementary table S1 Effect size of VAI on SUI in prespecified and exploratory subgroups.**

|  | OR (95%CI) | P for interaction |
| --- | --- | --- |
| age |  | 0.3835 |
| ≤50 | 1.07 (1.03, 1.12) |  |
| >50 | 1.05 (1.00, 1.10) |  |
| Race/Ethnicity |  | 0.7322 |
| Mexican American | 1.10 (1.02, 1.19) |  |
| Other Hispanic | 1.02 (0.94, 1.12) |  |
| Non-Hispanic White | 1.06 (1.02, 1.10) |  |
| Non-Hispanic Black | 1.10 (1.00, 1.22) |  |
| Other Race | 1.07 (0.92, 1.24) |  |
| Marital status |  | 0.6621 |
| Never married | 1.11 (0.97, 1.27) |  |
| Married or living with partner | 1.05 (1.01, 1.09) |  |
| Separated/Divorced/Widowed | 1.07 (1.02, 1.12) |  |
| Education |  | 0.6528 |
| Less than high school | 1.06 (1.01, 1.10) |  |
| Greater than high school | 1.07 (1.02, 1.13) |  |
| Insurance |  | 0.3995 |
| No | 1.04 (0.98, 1.10) |  |
| Yes | 1.07 (1.03, 1.11) |  |
| PIR |  | 0.0847 |
| <2 | 1.05 (1.02, 1.08) |  |
| ≥2 | 1.09 (1.05, 1.14) |  |
| Alcohol intake, g/d |  | 0.7657 |
| 0 | 1.07 (1.02, 1.12) |  |
| >0 | 1.04 (0.98, 1.11) |  |
| Caffeine intake, mg/d |  | 0.4418 |
| 0 - 40.5 | 1.10 (1.02, 1.18) |  |
| 41 - 141.5 | 1.09 (1.02, 1.17) |  |
| >141.5 | 1.03 (0.99, 1.08) |  |
| Physical activity, MET-min/week |  | 0.1932 |
| <150 | 1.05 (0.96, 1.16) |  |
| 150-960 | 1.09 (1.01, 1.17) |  |
| 961-1800 | 1.12 (1.01, 1.24) |  |
| >1800 | 1.09 (1.01, 1.18) |  |
| Diabetes |  | 0.067 |
| No | 1.09 (1.04, 1.13) |  |
| Yes | 1.03 (0.98, 1.08) |  |
| Hypertension |  | 0.0980 |
| No | 1.09 (1.04, 1.14) |  |
| Yes | 1.03 (0.98, 1.08) |  |
| GFR, mL/min/1.73 m^2^ |  | 0.0043 |
| <60 | 0.96 (0.89, 1.03) |  |
| ≥60 | 1.08 (1.03, 1.12) |  |
| Smoke |  | 0.4342 |
| Never | 1.06 (1.01, 1.12) |  |
| Former | 1.10 (1.02, 1.19) |  |
| Now | 1.04 (0.99, 1.10) |  |
| Parity |  | 0.2107 |
| 0 | 1.3 (0.9, 1.9) |  |
| 1 | 1.1 (1.0, 1.2) |  |
| 2 | 1.0 (1.0, 1.1) |  |
| ≥3 | 1.0 (1.0, 1.1) |  |
| PHQ-9 |  | 0.8558 |
| <10 | 1.07 (1.02, 1.12) |  |
| ≥10 | 1.05 (0.96, 1.14) |  |
| Comorbidity index |  | 0.3938 |
| 0 | 1.08 (1.02, 1.14) |  |
| 1 | 1.03 (0.98, 1.09) |  |
| ≥2 | 1.07 (1.02, 1.13) |  |

VAI-visceral adipose index; SUI-Stress Urinary Incontinence; PIR-poverty income ratio; GFR-Glomerular filtration rate; PHQ-9-Patient Health Questionnaire-9; CI-confidence interval; OR-odds ratio.
